# Assessment of coronary artery disease during hospitalization for cancer treatment

**DOI:** 10.1007/s00392-020-01719-5

**Published:** 2020-08-02

**Authors:** Simone M. Mrotzek, Alessia Lena, Sara Hadzibegovic, Ria Ludwig, Fadi Al-Rashid, Amir A. Mahabadi, Raluca I. Mincu, Lars Michel, Laura Johannsen, Lena Hinrichs, Martin Schuler, Ulrich Keller, Stefan D. Anker, Ulf Landmesser, Tienush Rassaf, Markus S. Anker, Matthias Totzeck

**Affiliations:** 1 Department of Cardiology and Vascular Medicine, West German Heart and Vascular Center, Medical Faculty, University Hospital Essen, Hufelandstr. 55, 45147 Essen, Germany; 2 Division of Cardiology and Metabolism, Department of Cardiology, Charité Campus Virchow Klinikum (CVK), Berlin, Germany; 3 Department of Cardiology, Charité Campus Benjamin Franklin (CBF), Berlin, Germany; 4 Berlin Institute of Health Center for Regenerative Therapies (BCRT), Berlin, Germany; 5 DZHK (German Center for Cardiovascular Research), Partner Site, Berlin, Germany; 6 Department of Medical Oncology, Medical Faculty, West German Cancer Center, University Hospital Essen, Hufelandstr. 55, 45147 Essen, Germany; 7 German Cancer Consortium (DKTK), Partner Site University Hospital Essen, Hufelandstrasse 55, 45147 Essen, Germany; 8 Department of Hematology, Oncology and Tumor Immunology, Charité Campus Benjamin Franklin (CBF), Berlin, Germany; 9 German Cancer Consortium (DKTK), Partner Site, Berlin, Germany; 10 Max-Delbrück Center for Molecular Medicine, Berlin, Germany

**Keywords:** Coronary artery disease, Acute coronary syndrome, Cancer, Cardio-oncology, Cardiotoxicity

## Abstract

**Background:**

With improvement of cancer-specific survival, comorbidities and treatment-related side effects, particularly cardiovascular toxicities, need close attention. The aim of the present study was to evaluate clinical characteristics and outcomes of cancer patients requiring coronary angiography during inpatient care.

**Methods:**

We performed a retrospective analysis of patients hospitalized between 02/2011 and 02/2018 in our two university hospital cancer centers. From a cohort of 60,676 cancer patients, we identified 153 patients (65.7 ± 11.6 years, 73.2% male), who underwent coronary angiography and were eligible for analysis. These were compared to a control group of 153 non-cancer patients pair-matched with respect to age, sex, and indication for catheterization.

**Results:**

Cancer patients presented in 66% with an acute coronary syndrome (ACS). The most prevalent cancer entities were lymphoma (19%) and lung cancer (18.3%). The rate of primary percutaneous coronary interventions (PCI) was significantly lower in the cancer cohort (40.5% vs. 53.6%, *p* = 0.029), although manifestation of coronary artery disease (CAD) and PCI results were comparable (SYNergy between PCI with TAXus and cardiac surgery (SYNTAX)-score, delta pre- and post-PCI − 9.8 vs. − 8.0, *p* = 0.2). Mortality was remarkably high in cancer patients (1-year mortality 46% vs. 8% in non-cancer patients, *p* < 0.001), particularly with troponin-positive ACS (5-year mortality 71%).

**Conclusion:**

Strategies to effectively control cardiovascular risks in cancer patients are needed. Additionally, suspected CAD in cancer patients should not prevent prompt diagnostic clarification and optimal revascularization as PCI results in cancer patients are comparable to non-cancer patients and occurrence of troponin-positive ACS leads to a significantly increased risk of mortality.

**Graphic Abstract:**

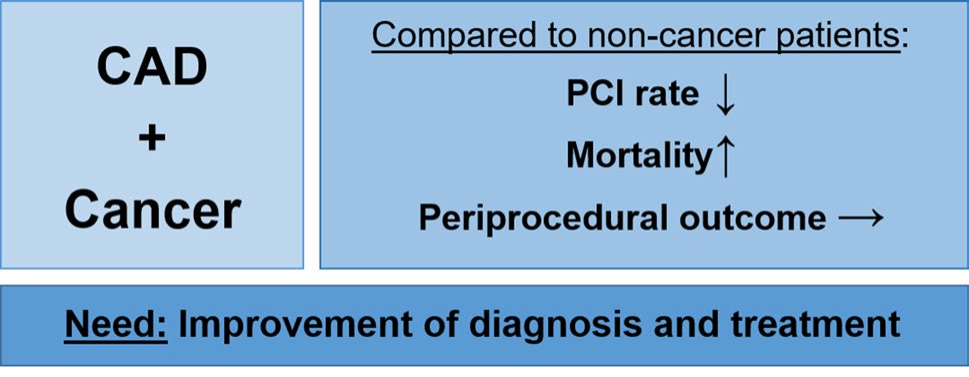

**Electronic supplementary material:**

The online version of this article (10.1007/s00392-020-01719-5) contains supplementary material, which is available to authorized users.

## Introduction

Cardiovascular diseases and cancer are the leading causes of death in western countries [1]. Cancer and cancer therapy may accelerate or induce coronary artery disease (CAD) and provoke acute coronary syndromes (ACS) [2]. Underlying mechanisms, treatment characteristics, and outcomes are incompletely characterized [3]. Additionally, common risk factors contribute to a higher prevalence of CAD in cancer patients [4]. Until now, guidelines for the treatment and management of patients with ACS and chronic coronary syndromes are not validated for cancer patients [5–7]. Uncertainties particularly pertain to the diagnosis of an ACS as biomarkers have been tested in only a limited number of cancer patients [8]. Troponin is as a well-established specific and sensitive marker of myocardial injury and infarction with high diagnostic and prognostic value for ACS patients [9–11]. In cancer patients, troponin release may additionally relate to anti-cancer treatments (e.g., anthracycline-related cardiotoxicity), which must be differentiated from an ACS [12–15]. Previous studies on patients with cancer and CAD showed that this cohort may be undertreated from the clinical and interventional point of view [16, 17]. Low platelet counts, bleeding complications, cancer surgery, percutaneous coronary intervention (PCI), and antiplatelet therapy represent major challenges in the treatment of cancer patients with CAD [18–20]. Decision algorithms for coronary angiography and peri-interventional management pathways for cancer patients are incompletely defined [3, 21]. Therefore, the objectives of our study were to evaluate clinical features and outcomes of this particular cancer cohort.

## Methods

**Fig. 1:**
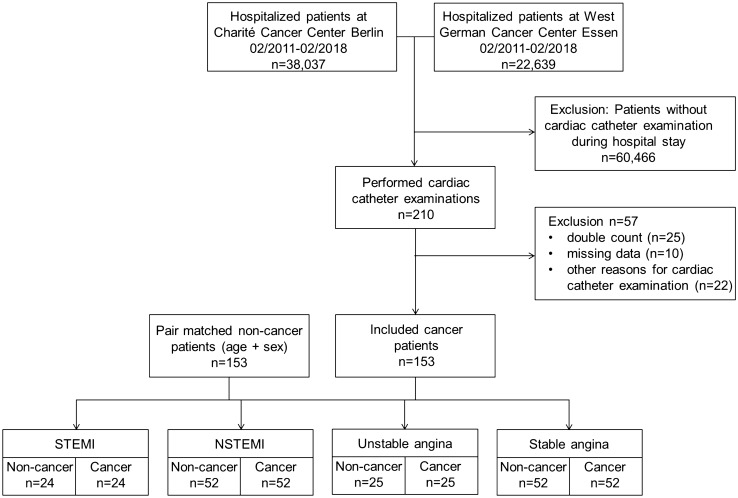
Flowchart of the study design. Medical data banks of patients hospitalized in the West German Cancer Center, University Hospital Essen and Charité Campus Benjamin Franklin Berlin between 02/2011 and 02/2108 were screened for performed coronary angiography using the clinical procedure code (OPS). From 60,676 patients, 210 patients were identified. 57 patients were excluded. Included cancer patients were divided by indication for coronary angiography: ST-segment elevation myocardial infarction (STEMI), non-STEMI (NSTEMI), unstable angina, and stable angina (including angina equivalents). Cancer patients were pair-matched with 153 non-cancer patients with respect to age, sex, and reason for coronary angiography

A retrospective, descriptive data analysis of patients of the West German Cancer Center, University Hospital Essen, and the Department of Hematology, Oncology and Tumor Immunology, Charité Campus Benjamin Franklin, Berlin (two of 13 comprehensive cancer centers in Germany), who were hospitalized between 02/2011 and 02/2018, was performed. In total, 60,676 patients were screened and all discharged patients with a clinical procedure code of coronary angiography (OPS 1-275, German adaption of the International Classification of Procedures in Medicine of the World Health Organization, version 2018) were reviewed. Screening identified 210 potentially eligible patients. After exclusion of 57 cases because of double count, missing data, or other reason for cardiac catheterization (e.g., cardiac biopsy and right heart catheterization), 153 patients treated at the cardiology departments of both hospitals [Department of Cardiology and Vascular Medicine, West German Heart and Vascular Center, University Hospital Essen and Department of Cardiology, Charité Campus Benjamin Franklin (CBF), Berlin, Germany] were eligible for further analyses. The study flowchart is illustrated in Fig. 1. Pair matching was performed using a “closest neighbor greedy” algorithm [22], to match each cancer and cancer-naive patient with respect to age (± 5 years), sex (male/female), and indication for coronary angiography. Patients were divided in four groups: ST-segment elevation myocardial infarction (STEMI), non-STEMI (NSTEMI), unstable angina, or stable angina (including patients with exertional angina pectoris and angina correlate). The non-cancer (control) cohort was randomly derived from patients hospitalized in the West German Heart and Vascular Center Essen or Department of Cardiology, Charité Campus Benjamin Franklin Berlin during 2017 and 2018 by reviewing reporting lists. The study was approved by the local ethics committees.

Statistical analyses of patient characteristics were conducted with respect to cancer entity, concurrent therapy, previous treatments, co-medication, and the individual patient’s medical history, especially known CAD and cardiovascular risk factors. Laboratory findings were compared as documented on the day before or day of performed coronary angiography. Bleeding complications in cancer and non-cancer patients have been analyzed referring to BARC (Bleeding Academic Research Consortium) definition [23]. A detailed assessment of cardiac catheter examination results was additionally performed. SYNTAX (SYNergy between percutaneous coronary intervention (PCI) with TAXus and cardiac surgery)-score calculation as a validated tool for risk stratification and revascularization strategy selection in patients with complex CAD [24, 25] was independently performed by two investigators. Patients with coronary artery bypasses were excluded [25]. SYNTAX-score values were classified according to the common standards in the lowest tertile (≤ 22), intermediate tertile (23–32), and highest tertile (≥ 33) [25]. PCI result was assessed by the residual SYNTAX-score and delta between pre- and post-PCI SYNTAX-score [26]. All-cause mortality of cancer patients was analyzed as documented in the database of our centers for a follow-up of up to 5 years after coronary angiography and 1 year for non-cancer patients.

Quantitative variables were checked for normal distribution and expressed as mean ± standard error of the mean (SEM) or as median and interquartile range (IQR, Q1 25% to Q3 75%). Dichotomous categorical variables were expressed as numeric values and percentages. For comparison of quantitative variables, an unpaired Student’s t test or Mann–Whitney *U* test (for non-normal distributed variables) was used. Dichotomous categorical variables were analyzed using Chi square test and Fisher’s exact test. Logistic regression analysis was used to determine the association of individual patients’ risk factors with PCI, presence of a multivessel CAD or 1-year mortality. Multivariable analyses were performed using the following adjustment sets: (1) age, gender, BMI, hypertension, diabetes, dyslipidemia, smoking, and previously known CAD adjusted, and (2) for cancer patients: age, gender, BMI, hypertension, diabetes, dyslipidemia, smoking, known CAD, metastatic disease, anthracycline chemotherapy, 5- fluorouracil chemotherapy, and chest irradiation adjusted, and (3) for 1-year mortality: age, gender, BMI, hypertension, diabetes, dyslipidemia, smoking, troponin-positive ACS, hemoglobin value, creatinine value, and platelet count. Kaplan–Meier procedure was calculated using log-rank tests (Mantel Cox) and Cox proportional hazard models to compare the survival curves of cancer and non-cancer patients as well as cancer patients with troponin-positive ACS (STEMI/NSTEMI) or troponin-negative presentation (unstable/stable angina). Five-year survival of cancer patients was additionally analyzed using data from the Surveillance, Epidemiology, and End Results Program (SEER) from the US National Cancer Institute. Cancer Statistics Review between 1975 and 2013 was released April 15, 2020 (https://seer.cancer.gov/archive/csr/1975_2013/results_merged/topic_survival.pdf) [27]. Listed survival rates from the SEER Cancer Statistics Review are calculated from 2006-2012. Patient’s data were matched to our study cancer cohort regarding sex, age, cancer entity, and the presence of a metastatic (distant) cancer disease. The statistical 5-year survival rate for each cancer patient from our study was determined from the SEER database and included as a part of the total 5-year survival rate for comparison with our cancer cohort. This was not possible for eight of our cancer patients due to missing information on the cancer entity within the SEER database (e.g., choroidal melanoma, choriocarcinoma) or not well-defined cancer entity (e.g., cancer of unknown origin).

Data were analyzed using IBM SPSS Statistics 25. Statistical significance was defined by a p value of < 0.05.

## Results

**Fig. 2:**
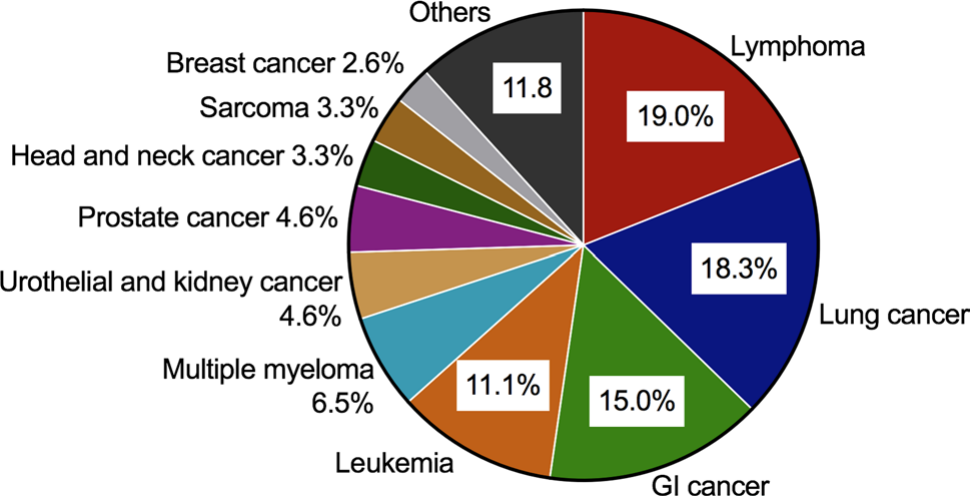
Differentiation of the study population by cancer entity. Lymphoma was the most common cancer entity with 29 patients (19%), followed by 28 patients with lung cancer (18.3%), 23 patients with gastrointestinal (GI) cancer (15%), and 17 patients with leukemia (11.1%)

In the present study, 153 cancer patients fulfilled the inclusion criteria and were pair-matched to 153 control patients without a history of cancer. Baseline characteristics of the study population are presented in Table 1. Differences between the groups regarding cardiovascular risk factors and medical history were found with a higher body mass index in non-cancer group (27.3 kg/m^2^ vs. 26.0 kg/m^2^, *p* = 0.014) and higher rates of documented dyslipidemia (66% vs. 48.4%, *p* = 0.003). Moreover, cancer patients had a higher occurrence of chronic obstructive pulmonary disease (11.8% vs. 20.9%, *p* = 0.044). Laboratory findings showed significantly lower hemoglobin value in cancer patients (13.62 g/dl vs. 10.83 g/dl, *p* < 0.001), as well as a trend to lower platelet counts (236/nl vs. 219/nl, *p* = 0.06). Previous treatment with acetylsalicylic acid or lipid-lowering therapy with statins was significantly less frequent in the cancer group (*p* = 0.001 and *p* < 0.001, respectively). Figure 2 illustrates the distribution of cancer entities in our study population. Lymphoma was the most common cancer entity with 29 patients (19%), followed by lung cancer (28 patients, 18.3%) and gastrointestinal cancer (23 patients, 15%).

**Table 1:** Baseline characteristics

	Non-cancer (*n *= 153)	Cancer (*n *= 153)	*p* value
Age, years	65.8 (± 11.8)	65.7 (± 11.6)	0.949
Male sex, *n* (%)	112 (73.2)	112 (73.2)	1.000
BMI, kg/m^2^	27.3 (± 4.6)	26.0 (± 4.8)	0.014*
Vascular risk factors, *n* (%)			
Diabetes mellitus	46 (30.1)	37 (24.2)	0.304
Smoking	61 (39.9)	62 (40.5)	1.000
Hypertension	126 (82.4)	121 (79.1)	0.562
Dyslipidemia	101 (66.0)	74 (48.4)	0.003*
Medical history, *n* (%)			
CAD	68 (44.4)	56 (36.6)	0.200
Atrial fibrillation	24 (15.7)	39 (25.5)	0.047*
PAOD	13 (8.5)	19 (12.4)	0.350
CAOD	11 (7.2)	8 (5.2)	0.637
Stroke	13 (8.5)	12 (7.8)	1.000
COPD	18 (11.8)	32 (20.9)	0.044*
Laboratory findings			
Trop pos., *n* (%)	73 (47.7)	74 (48.4)	1.000
Creatinine, mg/dl	1.08 (0.93–1.31)	1.06 (0.84-1.28)	0.056
GFR < 60 ml/min/1.73 m^2^, *n* (%)	55 (35.9)	62 (40.5)	0.480
Hb, g/dl	13.62 (± 2.05)	10.83 (± 1.79)	< 0.001*
Platelet count,/nl	236 (192–277)	219 (169-281)	0.060
Reduced EF (< 40%), *n* (%)	16 (12.3)	27 (18.8)	0.183
Medication history, *n* (%)			
Acetylsalicylic acid	99 (64.7)	70 (45.8)	0.001*
Dual antiplatelet therapy	17 (11.1)	15 (9.8)	0.852
Anticoagulation	16 (10.5)	14 (9.2)	0.848
Statin use	75 (49.0)	44 (28.8)	< 0.001*
Beta-blockers	82 (53.6)	97 (63.4)	0.104

*BMI* body mass index, *CAD* coronary artery disease, *PAOD* peripheral artery occlusive disease, *CAOD* cerebral artery occlusive disease, *COPD* chronic obstructive pulmonary disease, *Trop pos.* Troponin positive, *GFR* glomerular filtration rate, *Hb* hemoglobin, *EF* ejection fraction

Data are shown as mean (± SD) or median (IQR)

*Statistically significant difference between non-cancer and cancer cohort

**Fig. 3:**
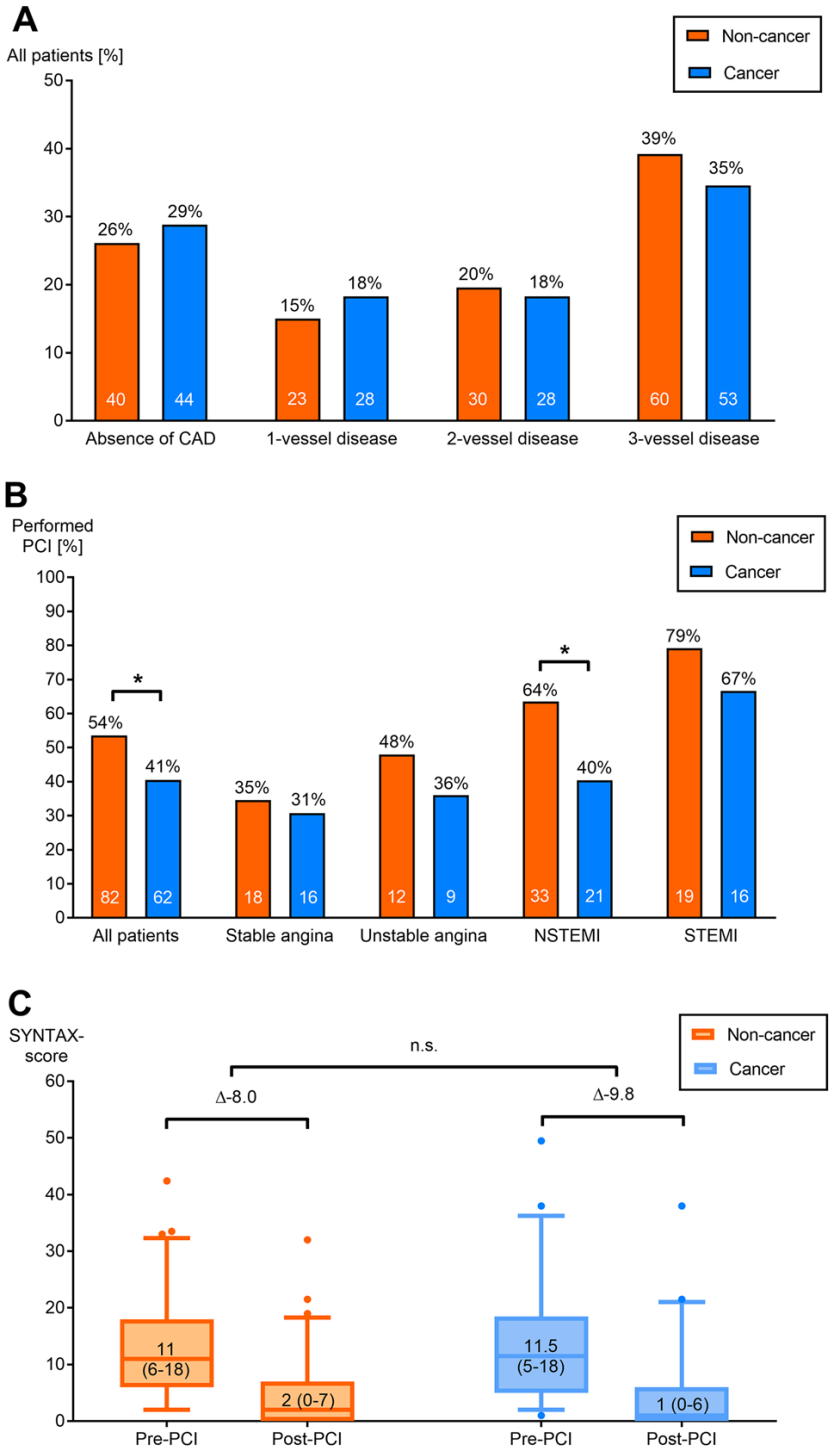
Results of cardiac catheter examinations. Analysis of coronary angiography results to distinguish between in 1-, 2-, and 3-vessel-disease was conducted. Occurrence of coronary artery disease (CAD) was similar in non-cancer and cancer patients (**a**). Rate of PCI related to clinical presentation showing a significant difference in non-cancer vs. cancer patients [all patients, (*p* = 0.029)], particularly with non-ST-segment elevation myocardial infarction (NSTEMI); *p* = 0.015 (**b**). Evaluation of coronary angiography results were conducted according to SYNTAX (SYNergy between PCI with TAXus and cardiac surgery)-score calculation. Patients with coronary artery bypasses were excluded. Improvement of SYNTAX-score after percutaneous coronary intervention (PCI) is illustrated before (pre-) and after (post-) PCI for non-cancer and cancer cohort (*p* = 0.2, not significant (n.s.)) (**c**)

Results of cardiac catheterization are summarized in Table 2. Obstructive CAD was not detectable in 26.1% of the non-cancer and in 28.8% of the cancer patients (*p* = 0.205). Figure 3a shows the occurrence of CAD and distinguishes between 1-, 2-, and 3-vessel-disease with no significant differences between the groups. Multivessel disease, defined as 2- or 3-vessel-disease, was found in 58.8% of the non-cancer and in 52.9% of the cancer patients. Rates of primary PCI are presented also in Fig. 3b. Cancer patients received a primary PCI less frequently (40.5% vs. 53.6%, cancer vs. non-cancer patients, *p* = 0.029). This difference could be traced back to patients presenting with NSTEMI (PCI rate 40.4% in cancer patients and 63.5% in non-cancer patients, *p* = 0.015), whereas PCI rate in patients with STEMI, unstable, or stable angina did not significantly differ between cancer and non-cancer groups. Multivariable logistic regression analysis identified especially dyslipidemia as a significant predictive factor for receiving a primary PCI in the cancer cohort (OR [95% CI]: 2.69 [1.211–5.973], *p* = 0.015), whereas, for example, smoking did not constitute a predictive factor in this context (0.906 [0.433–1.897], *p* = 0.794). For non-cancer patients, both dyslipidemia and smoking represent predictive factor for receiving a primary PCI (2.281 [1.074–4.845], *p* = 0.032 and 2.363 [1.147–4.868], *p* = 0.02, respectively). The presence of a multivessel CAD was detected more often in cancer patients with previously known CAD (12.9 [4.647-35.813], *p* < 0.001) and in non-cancer patients with diabetes (6.221 [2.099–18.435], *p* = 0.001), dyslipidemia (4.148 [1.691–10.172], *p* = 0.002), and also with previously known CAD (6.055 [2.509–14.612], *p* < 0.001). Regarding cancer history and therapy, the categories in univariate analysis (radiation, chemotherapy, anthracyclines, 5-fluorouracil, surgery, metastatic cancer, and cancer entity) had no significant impact on PCI rates (Supplemental Figure 1). Interestingly, multivariable analysis identified metastatic cancer and anthracycline chemotherapy as positive predictors for receiving a primary PCI (3.265 [1.455–7.325], *p* = 0.004 and 3.139 [1.228–8.027], *p* = 0.017). Treatment with 5-fluorouracil did not influence PCI rate significantly (0.425 [0.157–1.152], *p* = 0.093). From 27 patients with 5-fluorouracil treatment, ten patients had no obstructive coronary artery disease (37%), which is not significantly different compared to all included cancer patients (*p* = 0.650). Vasospasm, which is a typical 5-fluorouracil side effect, was suspected in only one case. Table 3 summarizes the results of cardiac catheter examination depending on chemotherapy with anthracyclines, 5-fluorouracil, and platinum-based or alkylating agents (e.g., cyclophosphamide).

**Table 2:** Results of cardiac catheter examinations

	Non-cancer (*n *= 153)	Cancer (*n *= 153)	*p* value
Findings, *n* (%)			
Multivessel disease	90 (58.8)	81 (52.9)	0.646
Absence of CAD	40 (26.1)	44 (28.8)	0.205
CTO	31 (10.3)	29 (19.0)	0.886
LMCA stenosis	10 (6.5)	18 (11.8)	0.164
Culprit lesion	84 (54.9)	74 (48.4)	0.303
Small vessel disease	68 (44.4)	65 (42.5)	0.818
PCI (any)	82 (53.6)	62 (40.5)	0.029*
DES	76 (49.7)	38 (24.8)	< 0.001*
BMS	1 (0.7)	24 (15.7)	< 0.001*
SYNTAX-score^†^			
Baseline-score	6 (0–13)	4 (0–12)	0.391
Lowest tertile (≤ 22), *n* (%)	127 (90.7)	122 (90.3)	1.000
Intermediate tertile (23–32)	7 (5.0)	9 (6.7)	0.613
Highest tertile (≥ 33)	6 (4.3)	4 (3.0)	0.750
Score post-PCI^‡^	0 (0–6)	0 (0–5)	0.578
Residual score > 8, *n* (%)	29 (20.7)	20 (14.8)	0.212
LVEDP^§^, mmHg	16 (12–20.75)	14 (8–20)	0.084

*CAD* coronary artery disease, *CTO* chronic total occlusion, *LMCA *= left main coronary artery, *PCI* percutaneous coronary intervention, *DES* drug-eluting stent, *BMS* bare-metal stent, *SYNTAX* SYNergy between PCI with TAXus and cardiac surgery, *LVEDP* left-ventricular end-diastolic pressure

Data are shown as median (IQR)

* Statistically significant difference between non-cancer and cancer cohort

^†^Excluded patients with history for coronary artery bypass surgery, included: cancer *n *= 135, non-cancer *n *= 140

^‡^Patients with performed PCI: cancer *n *= 57, non-cancer *n *= 73

^§^Documented LVEDP: cancer *n *= 110, non-cancer *n *= 102

**Table 3:** Results of cardiac catheter examinations depending on cancer therapy

	Anthracyclines(*n *= 35)	5-Fluorouracil (*n *= 27)	Platinum-based (*n *= 40)	Alkylating agents (*n *= 26)	All cancer patients (*n *= 153)
Findings, *n* (%)					
Multivessel disease	16 (45.7)	12 (44.4)	20 (50.0)	11 (42.3)	81 (52.9)
Absence of CAD	11 (31.4)	10 (37.0)	11 (27.5)	10 (38.5)	44 (28.8)
CTO	6 (17.1)	3 (11.1)	7 (17.5)	3 (11.5)	29 (19.0)
LMCA stenosis	5 (14.3)	2 (7.4)	2 (5.0)	3 (11.5)	18 (11.8)
Culprit lesion	19 (54.3)	13 (48.1)	21 (52.5	12 (46.2)	74 (48.4)
Small vessel disease	14 (40.0)	9 (33.3)	20 (50.0)	15 (57.7)	65 (42.5)
PCI (any)	16 (45.7)	8 (29.6)	19 (47.5)	13 (50.0)	62 (40.5)
DES	6 (17.1)	4 (14.8)	12 (30.0)	6 (23.1)	38 (24.8)
BMS	10 (28.6)*	4 (14.8)	7 (17.5)	7 (26.9)	24 (15.7)
SYNTAX-score^†^					
Baseline-Score	3 (0.0–7.75)	3.5 (0.0–7.75)	5 (0.0–13.88)	2 (0.0–7.0)	4 (0–12)
LVEDP^§^, mmHg	10 (7.0–15.0)	10 (7.25–15.5)	12 (7.25–22.25)	14.5 (7.75–22.75)	14 (8–20)

*CAD* coronary artery disease, *CTO* chronic total occlusion, *LMCA *= left main coronary artery, *PCI* percutaneous coronary intervention, *DES* drug-eluting stent, *BMS* bare-metal stent, *SYNTAX* SYNergy between PCI with TAXus and cardiac surgery, *LVEDP* left-ventricular end-diastolic pressure

Data are shown as median (IQR)

*Statistically significant difference between cancer patients under anthracycline therapy and all cancer patients

^†^excluded patients with history for coronary artery bypass surgery

^§^patients with documented LVEDP

Detailed evaluation of coronary angiography results was conducted according to the SYNTAX-scoring system (Table 2). Patients with coronary artery bypasses were excluded [25]. Baseline and proportion with a residual SYNTAX-score > 8 (defining an incomplete revascularization [26]) did not significantly differ between cancer and non-cancer patients. Change of SYNTAX-score upon PCI is depicted in Fig. 3c. No significant differences between cancer and control groups were found regarding pre-PCI and post-PCI SYNTAX-score (*n *= 73 non-cancer patients, *n *= 57 cancer patients). Delta between pre- and post-PCI SYNTAX-score as a marker for the PCI result was similar (non-cancer patients − 8.0, cancer patients -9.8 points, *p* = 0.2). Left-ventricular end-diastolic pressure as a marker for coronary microvascular disease was also similar in cancer cohort vs. non-cancer cohort (Table 2). However, differences were detected regarding peri-procedural device application: cancer patients received bare-metal stents more frequently (*p* < 0.001) and fewer drug-eluting stents (*p* < 0.001) compared to control group (Table 2).

Hemoglobin value was lower in cancer patients before coronary angiography (10.83 ± 1.79 g/dl vs. 13.62 ± 2.05 g/dl, *p* < 0.001), but hemoglobin drop within the first 48 h after coronary intervention did not differ significantly (median [IQR]: 0.55 [0.0–1.08] g/dl vs. 0.6 [0.0–1.40] g/dl, *p* = 0.329). Cancer patients received red blood cell transfusion more often (9.2% vs. 1.3%, *p* = 0.003), which was not linked to the occurrence of increased post-interventional bleeding or hematoma. Referring to the BARC definition of peri-interventional bleeding complications, hemoglobin drop between 3 and 5 g/dl (BARC 3a) occurred two times and > 5 g/dl (BARC 3b) once in both, cancer and control, groups. Surgical intervention (BARC 3b) was necessary once within the cancer group due to a femoral dissection. In-hospital mortality and 30-day mortality were not significantly different in cancer and non-cancer patients (HR 1.2 [0.51–2.76], *p* = 0.68), which indicates that acute complications or early cardiovascular events after cardiac catheterization (e.g., fatal bleeding and re-infarct rate) leading to death are similar in the two groups.

**Fig. 4:**
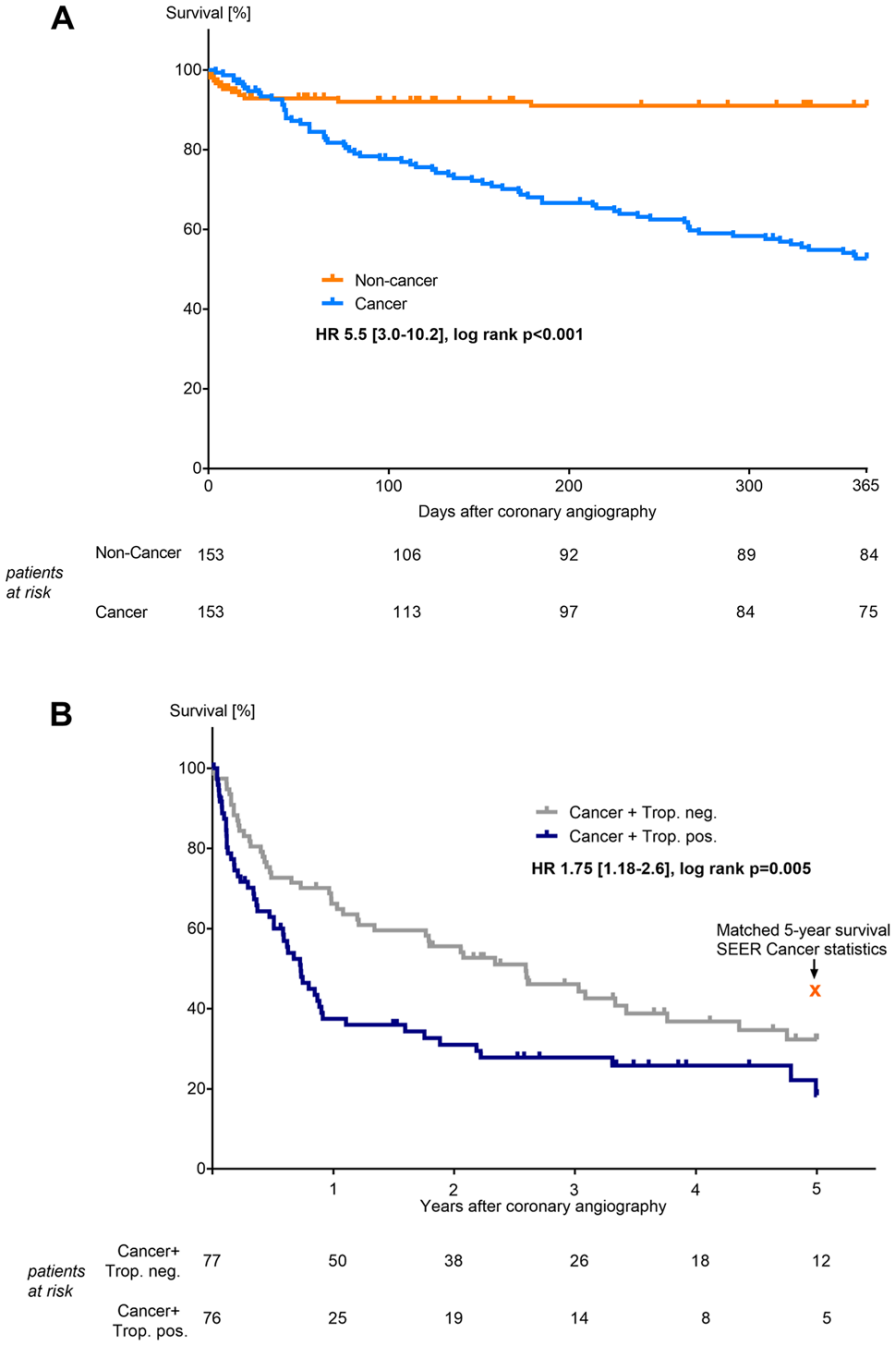
Kaplan–Meier analyses. One-year all-cause mortality of cancer patients was significantly higher compared to non-cancer patients, log-rank (Mantel Cox) *p* < 0.001. Mortality after 30 days was similar, log-rank (Mantel Cox) *p* = 0.68 (**a**). Five-year all-cause mortality of cancer patients with troponin-positive acute coronary syndrome (trop. pos./cancer patients with STEMI and NSTEMI) was significantly higher compared to cancer patients with troponin-negative angina (trop. neg./cancer patients with unstable and stable angina), log-rank (Mantel Cox) *p* = 0.005. Matched 5-year survival of cancer patients from the US National Cancer Institute Surveillance, Epidemiology, and End Results Program (SEER) was 42.4% (b)

All-cause mortality was analyzed using Kaplan–Meier procedure with a follow-up of 1 year showing a higher mortality in cancer patients compared to non-cancer patients (HR 5.5 [3.0–10.2], *p* < 0.001, Fig. 4a). Multivariable regression analysis identified male sex (0.286 [0.116-0.705], *p* = 0.007), troponin-positive ACS (2.365 [1.162–4.817], *p* = 0.018), and lower hemoglobin value (0.719 [0.575–0.898], *p* = 0.004) as predictors for 1-year mortality in cancer cohort. In non-cancer patients, none of the analyzed factors predicted 1-year mortality. Using Kaplan–Meier procedure, cancer patients with troponin-positive ACS (STEMI and NSTEMI) showed significantly higher 5-year mortality compared to cancer patients with troponin-negative angina (HR 1.75 [1.18–2.6], *p* = 0.005, Fig. 4b). Subgroup analysis between advanced cancer and non-advanced cancer did not abrogate this effect, showing the highest 5-year mortality in patients with advanced cancer and troponin-positive ACS (HR 1.59 [1.07-2.38], *p* = 0.023, Supplemental Figure 2). Advanced cancer was defined as metastatic cancer or depending on tumor stage (UICC stage III/IV, Ann-Arbor stage IV, Durie and Salmon stage 3, TMN stadium > T2b). Baseline characteristic of cancer patients with troponin-positive ACS vs. troponin-negative angina did not significantly differ (Supplemental Table 1), whereas results of cardiac catheter examinations documented a higher SYNTAX-score at baseline and a higher rate for PCI in cancer patients with troponin-positive ACS (Supplemental Table 2). To better classify the mortality rate of our study patients, we compared these with the 5-year survival of cancer patients from the US National Cancer Institute registry (SEER) [27]. Calculated 5-year survival rate based on SEER data would be 42.4%, which is not significantly higher compared to overall survival of 34% in our cancer cohort (*p* = 0.158, Fig. 4b). However, 5-year survival rate of cancer patients with troponin-positive ACS was with 28.9% significantly lower than in the calculated SEER cancer group (*p* = 0.017). This indicates that performing a coronary angiography in itself does not increase mortality in cancer patients, but the occurrence of a troponin-positive acute coronary syndrome may be associated with a higher mortality.

## Discussion

The concurrent incidence of cancer and CAD relates to overlapping risk factors, side effects of cancer therapy, and negative cardiovascular effects of cancer itself [4, 28]. Because of the high morbidity and mortality, cancer is often considered as a contraindication for coronary intervention [16, 17]. Patients with discharge diagnosis of STEMI or NSTEMI from a single-center assessment between 2000 and 2006 showed a remarkably low catheter-based revascularization rate of only 3.3%, because non-interventional therapy is often preferred in cancer patients [17]. In this study, we included patients assessed by coronary angiography during intra-hospital cancer treatment and conducted a comparison to a matched control cohort. The severity of CAD defined by the SYNTAX-score and distribution in 1-, 2-, or 3-vessel-disease did not differ significantly in cancer and non-cancer. However, the rate of PCI was lower in cancer patients, especially in patients with an NSTEMI diagnosis.

Our study confirms the results of previous reports, which highlighted a potentially insufficient treatment of patients with cancer and CAD from the clinical and interventional point of view [16, 17, 29–31] that was less adherent to American and European Guidelines [5, 6, 32]. Bare-metal stents have been preferred in cancer patients [18, 33], whereas data in general population unequivocally favor drug-eluting stents [34, 35]. This could have changed due implementation of new guidelines which no longer recommend these devices (31) and propose the same duration of dual antiplatelet therapy for 6 month for stable angina/chronic coronary syndromes and 12 months for ACS (for patients without indication for oral anticoagulation) for drug-eluting stents or drug-coated balloons [36]. However, detailed evidence for device selection and antiplatelet therapy is scarce. Interestingly, a large data analysis from the *National Inpatient Sample for Hospital Discharges* in the United States of America between 2004 and 2014 showed better outcomes in cancer patients and PCI when treated with drug-eluting stents rather than a bare-metal stents [31]. Bleeding complications are feared in cancer patients also because of lower hemoglobin values and platelet counts before coronary angiography. Application and duration of dual antiplatelet therapy pose a challenge, too, because cut-off values of low thrombocyte levels under cancer and cancer therapy do not exist coherently [30]. Additionally, thromboembolic events are frequently encountered in cancer patients, which can be treated by low-molecular-weight heparin [37] or oral anticoagulation with an acceptable incidence of major bleedings [38–41], but this complicates the therapeutic management in context of coronary angiography, PCI, and antiplatelet therapy. In-hospital and 30-day all-cause mortality as well as documented catheter-associated bleeding complications did not differ between cancer and non-cancer groups, which suggests a comparable peri-procedural risk in our study cohort.

In recent years, advances in tumor therapy lead to an improved survival of cancer patients [2, 42] and, therefore, it is important to emphasize the management of comorbidities and treatment-related cardiovascular toxicities [7, 43]. This also includes optimal primary and secondary prevention of cardiovascular diseases in cancer patients. The importance for early and intensive modification of cardiovascular risk factors was also highlighted in the last clinical practice guidelines from the American Society of Clinical Oncology for the treatment of breast cancer patients [44]. In our study population, previous treatment with acetylsalicylic acid and lipid-lowering therapy with statins was significantly less frequent in the cancer group, although there were no differences regarding patient’s medical history of known CAD and cardiovascular risk factors between cancer and control group.

The present study has several limitations. This study was retrospective and cancer patients were searched from the inpatient hospital database. Time interval between performed cancer therapy, whether chemotherapy or radiation, was not analyzed. Also, therapy protocols differ severely between the cancer patients. Overall, our study population is a mixed cohort but, therefore, representative for clinical practice. Differences between the cancer and non-cancer groups could have been influenced by different time periods of performed coronary angiography. Cancer patients were included between 2011 and 2018, whereas matched control patients received coronary angiography between 2017 and 2018. Management of patients with ACS or stable angina as well as myocardial revascularization strategies could be changed due to renewed practice guidelines [6, 9]. This, however, would not apply to the increased mortality in the cancer group itself. Selection of inpatient cancer patients comes along with high morbidity and mortality [45]. More than half of all cancer deaths occurred in an acute care hospital [46]. This leads to a comparably high 5-year mortality in our cancer cohort (66%), especially in patients with troponin-positive ACS (71%). This was also true in others studies with a 1-year survival as low as 26% of hospitalized cancer patients with ACS [17]. Another study documented a significant greater in-hospital mortality of patients with lung cancer undergoing PCI [31], which was one of the most common cancer entities in our study population. Our data only provide information of all-cause mortality. Determining the cause of death is usually difficult in cancer patients due to a complex clinical picture of existing diseases, especially within the last weeks before death. Often palliative care is needed because of pain medication and sedation. Clinical studies and publications dealing with this topic are rare. Different cancer entities seem to be associated with different causes of death [47]. Multiorgan failure, cardiovascular diseases, cachexia, and severe infection are some of the most named causes of death in cancer patients [48, 49]. To avoid any classification bias, we focused on all-cause mortality in this study.

## Conclusions

Hospitalized cancer patients were undertreated regarding performed PCI, particularly patients with troponin-positive ACS which additionally showed the highest mortality rate. SYNTAX-scores suggest a good interventional result in cancer patients if PCI was performed. This underlines the importance of further characterization and recommendation for clinical behavior and treatment of these patients. Initial management of cancer patients with elevated troponin or angina pectoris symptoms remains difficult in daily clinical practice. Identification of cardiovascular risk factors, and primary and secondary prevention are highly important especially in cancer patients. Cardio-oncology is a multidisciplinary field with increasing patient numbers and, therefore, poses a challenge to the medical system.

## Electronic supplementary material

Below is the link to the electronic supplementary material.

Supplementary material 1 (PDF 326 kb)

## Data Availability

The datasets generated and/or analyzed during the current study are available from the corresponding author upon reasonable request.
