# Supplementary material for: Assessment of coronary artery disease during hospitalization for cancer treatment
Source: Clin Res Cardiol. 2020 Aug 2;110(2):200–10. doi: 10.1007/s00392-020-01719-5 (PMC7862534; doi:10.1007/s00392-020-01719-5)
Supplement: Supplementary file 1 — Supplementary material 1 (PDF 326 kb) [file 392_2020_1719_MOESM1_ESM.pdf]

Assessment of coronary artery disease during hospitalization for cancer treatment

Simone M. Mrotzek[1], Alessia Lena[2,3,4,5], Sara Hadzibegovic[2,3,4,5], Ria Ludwig[1], Fadi Al-Rashid[1], Amir A. Mahabadi[1], Raluca I. Mincu[1], Lars Michel[1], Laura Johannsen[1], Lena Hinrichs[1], Martin Schuler[6,7], Ulrich Keller[8], Stefan D. Anker[2,4,5], Ulf Landmesser[3,4,5], Tienush Rassaf[1], Markus S. Anker[2,3,4,5], Matthias Totzeck[1]

[1]Department of Cardiology and Vascular Medicine, West German Heart and Vascular Center, Medical Faculty, University Hospital Essen Hufelandstr. 55, 45147 Essen, Germany

[2]Division of Cardiology and Metabolism, Department of Cardiology, Charité Campus Virchow Klinikum (CVK), Berlin, Germany

[3]Department of Cardiology, Charité Campus Benjamin Franklin (CBF), Berlin, Germany

[4]Berlin Institute of Health Center for Regenerative Therapies (BCRT), Berlin, Germany

[5]DZHK (German Center for Cardiovascular Research), partner site Berlin, Germany

[6]Department of Medical Oncology, West German Cancer Center, Medical Faculty, University Hospital Essen, Hufelandstr. 55, 45147 Essen, Germany

[7]German Cancer Consortium (DKTK), Partner site University Hospital Essen, Hufelandstrasse 55, 45147 Essen, Germany

[8]Department of Hematology, Oncology and Tumor Immunology, Charité Campus Benjamin Franklin (CBF), Berlin, Germany

Corresponding author:
Dr. Matthias Totzeck
Department for Cardiology and Vascular Medicine
West German Heart and Vascular Center
Medical Faculty, University Hospital Essen
Hufelandstraße 55, 45147 Essen, Germany
Phone: +49 201 723 4818
Fax: +49 201 723 5401
Matthias.Totzeck@uk-essen.de

Supplemental fig. 1

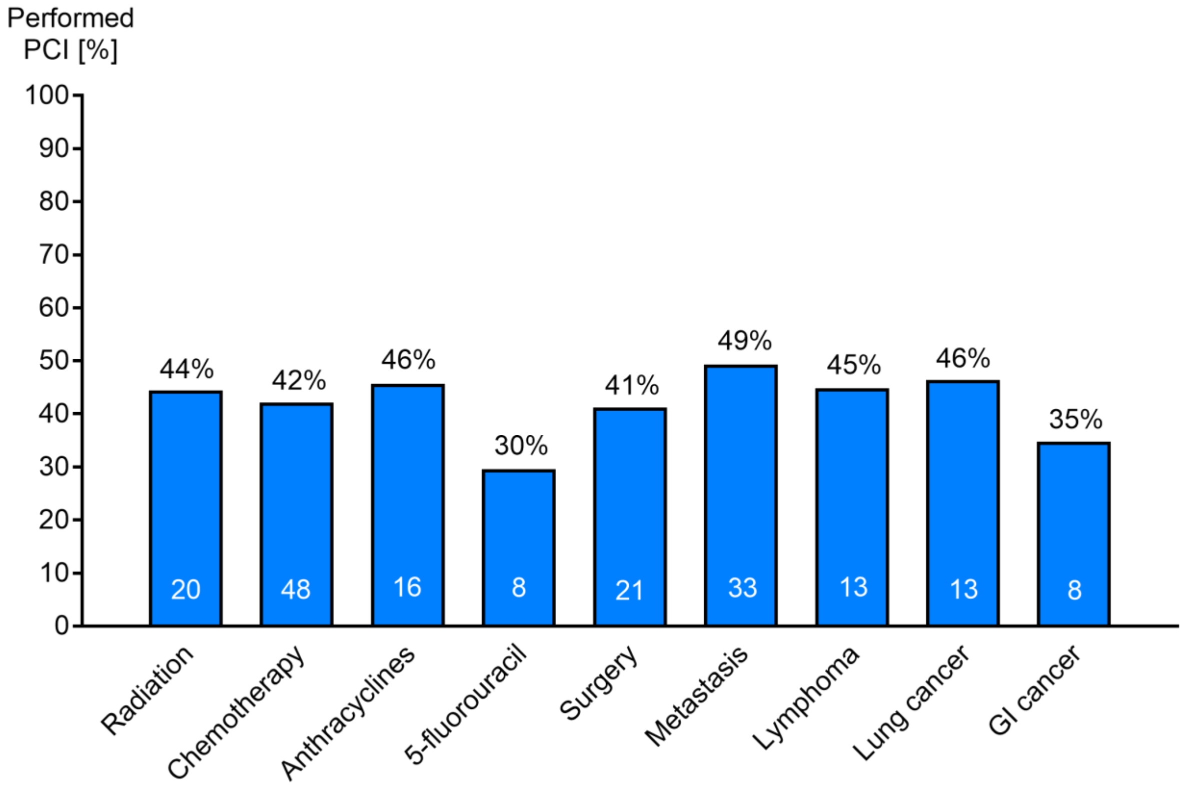

Rate of performed primary percutaneous coronary intervention (PCI) related to cancer therapy and entity showing no significant differences between groups.

Supplemental fig. 2

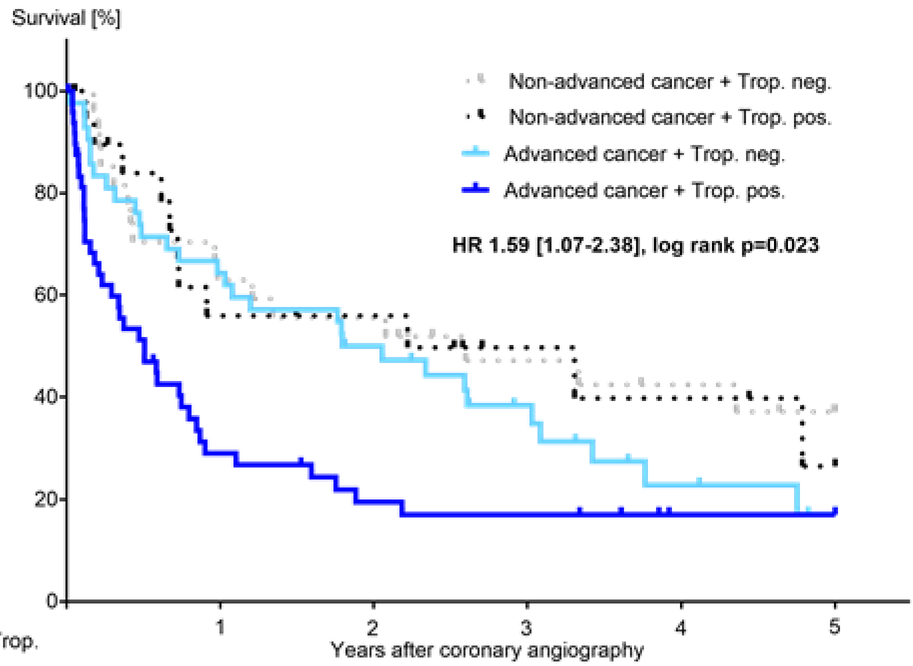

	Cancer	Trop.		1	2	3	4	5
patients at risk	Non-advanced	neg.	27	17	15	10	8	5
	Non-advanced	pos.	21	10	9	5	4	2
	Advanced	neg.	42	27	18	11	5	2
	Advanced	pos.	51	13	8	7	3	3

Five year all-cause mortality of cancer patients divided in advanced and non-advanced cancer with troponin positive acute coronary syndrome (trop. pos./ STEMI and NSTEMI) or troponin negative angina (trop. neg./ unstable and stable angina). Patients with advanced cancer presented with trop. pos. ACS had the highest mortality, log rank (Mantel Cox) p=0.023.

Supplemental table 1

Baseline characteristics of cancer patients with troponin-positive acute coronary syndrome (trop. pos.) vs. troponin-negative angina (trop. neg.)

	**Cancer + Trop. neg. (n=77)**	**Cancer + Trop. pos. (n=76)**	***P* value**
**Age, years**	67.1 (± 11.4)	64.42 (± 11.5)	0.160
**Male sex, n (%)**	55 (71.4)	57 (75)	0.716
**BMI, kg/m²**	26.4 (± 4.8)	25.6 (± 4.8)	0.300
**Vascular risk factors, n (%)**			
**Diabetes mellitus**	19 (24.7)	18 (23.7)	1.000
**Smoking**	30 (39.0)	32 (42.1)	0.743
**Hypertension**	63 (81.8	58 (76.3)	0.432
**Dyslipidemia**	37 (48.1)	37 (48.7)	1.000
**Medical history, n (%)**			
**Coronary artery disease**	32 (41.6)	24 (31.6)	0.241
**Atrial fibrillation**	23 (29.9)	16 (21.1)	0.266
**PAOD**	8 (10.4)	11 (14.5)	0.473
**CAOD**	2 (2.6)	6 (7.9)	0.167
**Stroke**	3 (3.9)	9 (11.8)	0.079
**COPD**	15 (19.5)	17 (22.4)	0.695
**Laboratory findings**			
**Creatinine, mg/dl**	1.03 (0.77-1.32)	1.08 (0.86-1.26)	0.900
**GFR <60 ml/min/1.73m², n (%)**	33 (42.9)	29 (38.2)	0.622
**Hb, g/dl**	11.1 (± 1.7)	10.6 (± 1.9)	0.300
**Platelet count, /nl**	221 (174-282)	215 (152-277)	0.600
**Reduced EF (<40%), n (%)**	11 (14.7)	16 (23.2)	0.207
**Medication history, n (%)**			
**Acetylsalicylic acid**	39 (50.6)	31 (40.8)	0.257
**Dual antiplatelet therapy**	11 (14.3)	4 (5.3)	0.100
**Anticoagulation**	8 (10.4)	6 (7.9)	0.780
**Statin use**	24 (31.2)	20 (26.3)	0.593
**Beta-blockers**	52 (67.5)	45 (59.2)	0.317

BMI body mass index, *PAOD* peripheral artery occlusive disease, *CAOD* cerebral artery occlusive disease, *COPD* chronic obstructive pulmonary disease, *GFR* glomerular filtration rate, *Hb* hemoglobin, *EF* ejection fraction

Data is shown as mean (± SD) or median (IQR)

Supplemental table 2

Results of cardiac catheter examinations in Cancer patients with troponin-positive acute coronary syndrome (trop. pos.) vs. troponin-negative angina (trop. neg.)

	**Cancer + Trop. neg. (n=77)**	**Cancer + Trop. pos. (n=76)**	***P* value**
**Findings, n (%)**			
**Multivessel disease**	37 (48.1)	44 (57.9)	0.258
**Absence of CAD**	26 (33.8)	18 (23.7)	0.212
**CTO**	11 (14.3)	18 (23.7)	0.154
**LMCA stenosis**	7 (9.1)	11 (14.5)	0.327
**Culprit lesion**	33 (42.9)	41 (53.9)	0.197
**Small vessel disease**	31 (40.3)	34 (44.7)	0.625
**PCI (any)**	25 (32.5)	37 (48.7)	0.049*
**DES**	12 (15.6)	26 (34.2)	0.009*
**BMS**	11 (14.3)	13 (17.1)	0.663
**SYNTAX-score†**			
**Baseline-Score**	2 (0-7)	7 (0-15.25)	<0.001*
**Lowest tertile (≤22), n (%)**	66 (97.1)	56 (83.6)	0.009*
**Intermediate tertile (23-32)**	2 (2.9)	7 (10.4)	0.096
**Highest tertile (≥33)**	0	4 (6.0)	0.058
**Score post PCI‡**	0 (0)	2 (0-2)	0.170
**Residual score >8, n (%)**	8 (11.8)	12 (17.9)	0.343
**LVEDP§, mmHg**	13 (5.25-16)	15 (7-22.75)	0.158

CAD coronary artery disease, *CTO* chronic total occlusion, *LMCA* left main coronary artery, *PCI* percutaneous coronary intervention, *DES* drug eluting stent, *BMS* bare metal stent, *SYNTAX* SYNergy between PCI with TAXus and cardiac surgery, *LVEDP* left ventricular end-diastolic pressure

Data is shown as median (IQR)

*****Statistically significant difference between Trop. neg. vs. Trop. pos. cancer patients

†excluded patients with history for coronary artery bypass surgery, included: Cancer + Trop. neg. n=68, Cancer + Trop pos. n=67

‡patients with performed PCI: Cancer + Trop. neg. n=21, Cancer + Trop pos. n=37

§ documented LVEDP: Cancer + Trop. neg. n=57, Cancer + Trop pos. n=53
